# Assessing the Stability of Polymer Inclusion Membranes: The Case of Aliquat 336-Based Membranes

**DOI:** 10.3390/membranes15100309

**Published:** 2025-10-13

**Authors:** Kalina Velikova, Todor Dudev, Tsveta Sarafska, Lea Kukoc-Modun, Spas D. Kolev, Tony Spassov

**Affiliations:** 1 Faculty of Chemistry and Pharmacy, Sofia University “St. Kl. Ohridski”, 1 James Bourchier Blvd., 1164 Sofia, Bulgaria; mjashkova@uni-sofia.bg (K.V.); ohtttd@chem.uni-sofia.bg (T.D.); nhtth@chem.uni-sofia.bg (T.S.); 2 Department of Analytical and Environmental Chemistry, Faculty of Chemistry and Technology, University of Split, Ruđera Boškovića 35, 21000 Split, Croatia; kukoc@ktf-split.hr; 3 School of Chemistry, The University of Melbourne, Melbourne, VIC 3010, Australia; 4 Department of Chemical Engineering, The University of Melbourne, Melbourne, VIC 3010, Australia; 5 National Centre of Excellence Mechatronics and Clean Technologies, 8 bul. Kliment Ohridski, 1164 Sofia, Bulgaria

**Keywords:** polymer inclusion membrane (PIM), Aliquat 336, stability, extractant leaching, thermal analysis, DFT

## Abstract

Leaching of the extractant from polymer inclusion membranes (PIMs) into the feed and receiving aqueous solutions shortens their life. Therefore, when a particular PIM extractant has been selected, it is important to choose a base polymer that will minimize to the greatest extent extractant leaching compared to other base polymers, thus providing the best stability of the PIM. However, comparisons of the stability of PIMs composed of the same extractant and different base polymers is usually conducted by multiple cycles of extraction and back-extraction steps, which are time-consuming and labor-intensive. An alternative approach based on thermal analysis (thermogravimetric analysis (TGA) and differential thermal analysis (DTA)) was developed and applied to PIMs containing 40 wt.% Aliquat 336, one of the most frequently used PIM extractants, and the three most frequently used PIM base polymers, i.e., poly(vinyl chloride) (PVC), cellulose triacetate (CTA), and poly(vinylidene fluoride-co-hexafluoropropylene) (PVDF-HFP). The temperatures and enthalpies associated with Aliquat 336 release were compared, with PVDF-HFP exhibiting the highest values, indicating the strongest interaction between the extractant and the polymer matrix and, thus, the highest stability. The PVC-based PIM was predicted to be the most prone to extractant leaching among the PIMs studied. This stability ranking was confirmed theoretically by quantum chemistry (DFT) calculations, which provided molecular-level insights into the likely interaction sites between Aliquat 336 and the polymer chains. An experimental validation of the above leaching order was also provided by PIM leaching experiments in aqueous 0.1 M and 0.05 M NaCl solutions, where membrane mass losses over a 24 h period were determined. The results of the current study demonstrated thermal analysis to be a fast and viable approach in comparing the stability of PIMs with the same extractant but different base polymers.

## 1. Introduction

Polymer inclusion membranes (PIMs) have attracted significant research interest in recent years as a promising green alternative to conventional solvent extraction, which uses significant amounts of often toxic, flammable, and volatile diluents [1,2,3]. A typical PIM is a thin flexible and homogeneous self-supporting film consisting of a base polymer (e.g., poly(vinyl chloride) (PVC), cellulose triacetate (CTA), poly(vinylidene fluoride), and its derivatives, such as poly(vinylidene fluoride-co-hexafluoropropylene) (PVDF-HFP)) and a commercial extractant (e.g., Aliquat 336 and di(2-ethylhexyl)phosphoric acid (D2EHPA)) located between the entangled chains of the base polymer. In most cases, the extractant acts as a plasticizer, but, in some cases, it might be necessary to use a separate plasticizer (e.g., 2-nitrophenyl octyl ether) or modifier (e.g., long-chain alcohols) to improve the compatibility between the base polymer and the extractant or to increase the solubility of the adduct between the target chemical species and the extractant in the membrane liquid phase [1,2]. Separation based on PIMs virtually eliminates the use of diluents and minimizes the amount of extractants required, thus making the utilization of highly selective but expensive extractants economically feasible. Another important advantage of PIMs is that the extraction of the target chemical species from a feed solution and its back-extraction into a receiving solution take place simultaneously at the corresponding membrane/solution interfaces. This simplifies the separation process compared to conventional solvent extraction, where extraction and back-extraction are usually conducted sequentially and allows for the development of continuous PIM-based separation processes. The fabrication of PIMs is usually based on dissolving the PIM components into a volatile (e.g., tetrahydrofuran for PVC- and PVDF-HFP-based PIMs and dichloromethane for CTA-based PIMs) or water soluble (e.g., tetrahydrofuran) solvent and pouring the solution into a cast or using a casting knife. The PIM is formed after the solvent is removed either by evaporation or by dissolution into water [4].

A PIM-based separation consists of three simultaneously occurring processes. One involves transporting the target chemical species (usually an ion) from the feed solution to the membrane surface where it forms an adduct with the extractant (e.g., ion pair or complex). Another one involves transporting the adduct across the membrane along the corresponding concentration gradient, and in the third process the target chemical species is stripped into a receiving solution as a result of a back-extraction reaction taking place at the membrane/receiving solution interface [1].

Due to their chemical composition, PIMs are mechanically strong, thermally stable and chemically resistant to strong acids. These properties, combined with their insignificant negative impact on the environment (i.e., relatively small consumption of reagents for their manufacturing and possibility for reuse), makes them attractive to various industrial separation processes (e.g., hydrometallurgy [5,6] and wastewater technology for removing pollutants [7,8]) and chemical analysis [9].

An important property of PIMs that determines to a significant extent their industrial applicability and supports their green credentials is their stability [1,2]. An adequate stability will allow a PIM to be used multiple times in batch separation processes or for extended periods of time in continuous separation processes. Membrane stability is determined mostly by the resistance of the membrane liquid phase to leaching into the adjacent feed and/or receiving solutions. PIMs are significantly more stable than supporting liquid membranes (SLMs), which are arguably the most frequently used liquid membranes at present [1]. The most likely reason for this difference is that unlike SLMs, where the membrane liquid phase is retained by relatively week capillary force in the micrometer size membrane pores, the membrane liquid phase in PIMs is located in a network of nanometer-sized channels [10]. Despite this fact, loss of PIM liquid phase takes place during both extraction and back-extraction due to extractant leaching, and it has been found that, in some cases, this may affect PIM morphology [11]. The magnitude of extractant loss is determined to a considerable extent by its solubility in the aqueous phases in contact with the PIM, which can be manipulated, to some extent, by adjusting their chemical composition (e.g., reducing pH in the case of acidic extractants such as D2EHPA) [2]. However, it can be expected that the chemical interactions between the extractant and the base polymer determine not only their compatibility and, thus, the possibility of forming a homogeneous PIM [12] but also the strength of extractant retention within the PIM and, therefore, PIM stability. While predicting the stability of PIMs with the same base polymer but different extractants could be conducted by comparing the water solubilities of the corresponding extractants, a similarly straightforward assessment involving PIMs incorporating the same extractant in different base polymer is not possible. FTIR spectra of PIMs (e.g., PIMs containing Aliquat 336 and PVC [13], CTA [14], or PVDF [15] as base polymers) do not show new peaks corresponding to covalent bonding between the extractant and the base polymer, only shifts in vibration frequencies of existing peaks attributed to weak interactions such as Van der Waals and dipole–dipole forces. However, quantifying these interactions based on these shifts is generally not possible. The conventional approach in studying the stability of a PIM is usually based on comparing its performance in repeated cycles of extraction and back-extraction using fresh feed and receiving solutions in each cycle (e.g., [16,17,18,19]) or monitoring its performance in a continuous flow-through membrane separation system over an extended period of time (e.g., [8,20]). However, this approach is time-consuming and labor intensive, which becomes even more pronounced when the stability of several PIMs is examined for selecting the most stable one [16]. This is the reason why most stability studies did not involve comparisons between the stability of PIMs with the same extractant but different base polymers [18]. In some cases, articles may report on the stability studies of PIMs with the same extractant but a different base polymer. However, the corresponding experimental conditions are different (e.g., Cr(VI) extraction by Aliquat 336 PIMs containing 2-nitrophenyl octyl ether as plasticizer and PVDF [15] and CTA [21] as base polymers), and it is not possible to use the stability data to reliably compare the base polymer’s effect on the membrane stability. Therefore, a novel approach allowing for comparison of the stability of PIMs containing the same extractant but different base polymers that is faster than conducting multiple extraction and back-extraction experiments is required.

The present paper reports on such an approach for relatively fast stability ranking of PIMs with the same extractant but different base polymers. It employs thermal analysis for assessing the strength of interactions between the PIM extractant and base polymer based on the temperature and energy of the extractant’s separation/desorption from the polymer matrix. The approach has been applied to PIMs incorporating Aliquat 336, one of the most frequently used extractants in PIMs that can form ion pairs with various anions [22], and PVC, CTA, or PVDF-HFP as the base polymer. The stability order obtained by thermal analysis has been supported by quantum chemical (DFT) calculations and leaching experiments involving the PIMs mentioned above.

The thermal analysis also allows for the assessment of the PIM’s thermal stability, which can affect their potential for use in separation processes at elevated temperatures such as various wastewater treatment processes [23].

## 2. Materials and Methods

### 2.1. Reagents

The following reagents, purchased from Merck/Sigma-Aldrich (Darmstadt, Germany), were used to prepare the PIMs studied: Aliquat 336, high-molecular-weight PVC, PVDF-HFP, CTA, tetrahydrofuran (THF, inhibitor-free), and dichloromethane (DCM). All solutions were prepared in deionized water (18 MΩ cm, Millipore, Synergy 185, Alsace, France).

### 2.2. Membrane Preparation

The PIMs used in this study contained 60 wt.% base polymer (PVC, CTA, or PVDF-HFP) and 40 wt.% Aliquat 336, which is a mixture of quaternary alkylammonium chlorides with the dominant species being N-methyl-N,N,N-trioctyl ammonium chloride. In their preparation, 240 mg of the base polymer and 160 mg of Aliquat 336 were dissolved in 15 mL of THF for the PVC- and PVDF-HFP-based PIMs and DCM for the CTA-based PIMs under stirring, as well as heating at 40 °C in the case of PVDF-HFP, on a mixing platform (MULTI-HS 6/15 Digital Multi-position Hot Plate Stirrer, VELP, Italy). Once the membrane components were completely dissolved, the solution was poured into a glass Petri dish, which was covered with filter paper and watch glass to slow down the evaporation of the solvent and allowed to stand for 24 h to allow for complete evaporation of the solvent.

### 2.3. PIM Leaching Studies

The mass loss due to Aliquat 336 leaching from 3 PIMs of each base polymer was measured after immersion of each PIM in 150 mL of 0.1 M NaCl and 0.05 M NaCl solutions for 24 h under stirring at room temperature.

### 2.4. Thermal Analysis

Thermal analysis techniques (thermogravimetric analysis (TGA) and differential thermal analysis (DTA)) were employed (simultaneous thermal analyzer SD650, Waters) to evaluate the thermal stability and degradation profiles of the PIMs studied. The thermal methods were used to assess the stability of Aliquat 336 within the polymer matrix, providing insight into potential decomposition temperatures and leaching tendencies. The thermal behavior of the membranes was investigated under controlled heating conditions (10 K/min, pure nitrogen atmosphere) to determine their thermal resistance and component loss characteristics. We used a standard (melting enthalpy of In) to calibrate the thermal effects.

### 2.5. Computational Methods

#### 2.5.1. Models Used

**Figure 1:**
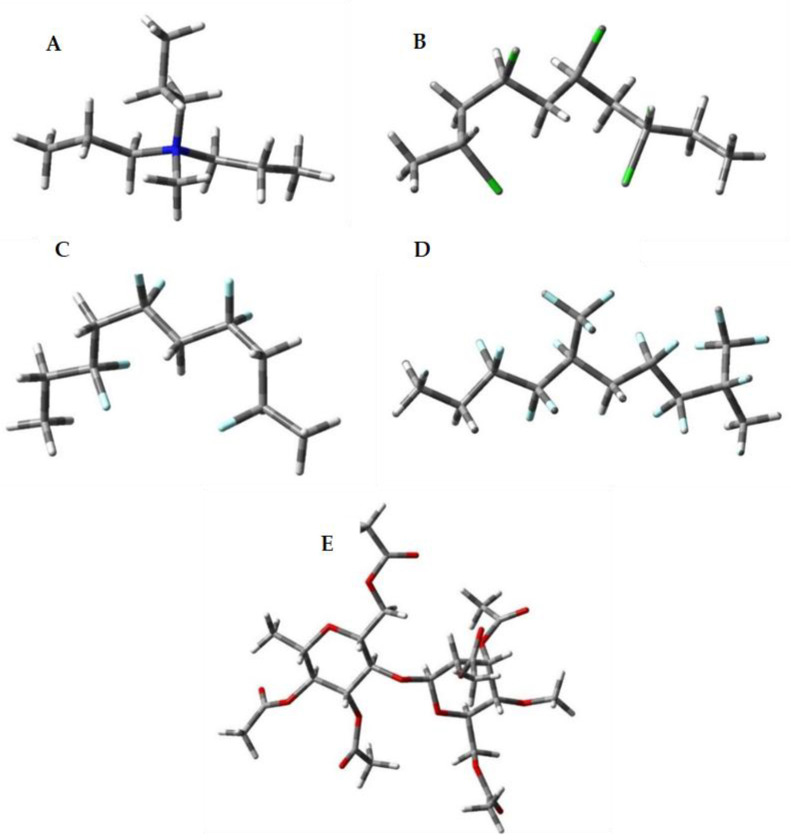
M062X/6-31+G(d,p) fully optimized structures of model (**A**) Aliquat 336 cation; (**B**) tetrameric PVC; (**C**) tetrameric “light” PVDF-HFP; (**D**) dimeric “heavy” PVDF-HFP; and (**E**) dimeric CTA molecules. Color scheme: carbon–dark gray, oxygen–red, chlorine–green, fluorine–pale blue, nitrogen–blue, and hydrogen–light gray.

The positively charged unit of Aliquat 336 (N-methyl-N,N,N-trioctyl ammonium) was modeled as N-methyl-N,N,N-tripropyl ammonium cation (for the sake of reducing the computer memory demand) as shown in Figure 1A. PVC and PVDF-HFP are represented as tetramers (Figure 1B–D). The following two types of structures were considered for PVDF-HFP as this is a co-polymer with varying content of “light” -CH_2_-CF_2_- and “heavy” -CF_2_-CF(CF_3_)- monomers: tetramer build up entirely of “light” monomers (Figure 1C) and of alternating “light”–“heavy”–“light”–“heavy” units (Figure 1D). CTA, containing bulky fully acetylated D-glucose fragments, was modeled as a dimer (Figure 1E).

#### 2.5.2. DFT Calculations

Density functional theory (DFT) computations on model Aliquat 336 cation and polymeric structures were performed. The geometries of all participating entities were optimized at the M062X/6-31+G(d,p) level of theory using the Gaussian 09 suite of programs [23]. The Minnesota M062X functional in combination with a split-valence double-ζ basis set was employed in the calculations, as it has been proven to be reliable in reproducing the geometrical parameters of a number of large organic systems and their complexes with organic/inorganic substances [24,25,26]. The optimized coordinates of the participating molecules/complexes, along with the convergence criteria, in each case are provided in the Supplementary Materials. It should be noted that dispersion corrections are automatically taken care of by employing the last generation of M062X functional. Moreover, this basis set represents a reasonable compromise between calculation accuracy and computer memory demands, which is an important factor to consider when dealing with large molecular structures. Electronic energies, E_el_, vibrational frequencies (all of them real), thermal energies, including zero-point energies, Eth, and entropies, S, at 25 °C and 1 atm pressure were evaluated at the same level of theory. Enthalpies, ΔH, and Gibbs free energies, ΔG, of the complex formation between the polymer host and Aliquat 336 cationic guest (Equation (1)) were calculated, taking into account differences in the respective quantities between the products and reactants of the process (Equations (2) and (3)).

*Aliquat 336 cation* + *Polymer* → [*Aliquat 336 cation*: *Polymer*] (1)

ΔH = ΔE_el_ + ΔEth − ΔpV (2)

ΔG = ΔH − TΔS(3)

In Equation (2), ΔpV is a work term accounting for the molar difference between the two arms of Equation (1) (0.59 kcal/mol in this case).

## 3. Results and Discussion

### 3.1. DTA/TG PIM Stability Study

**Figure 2:**
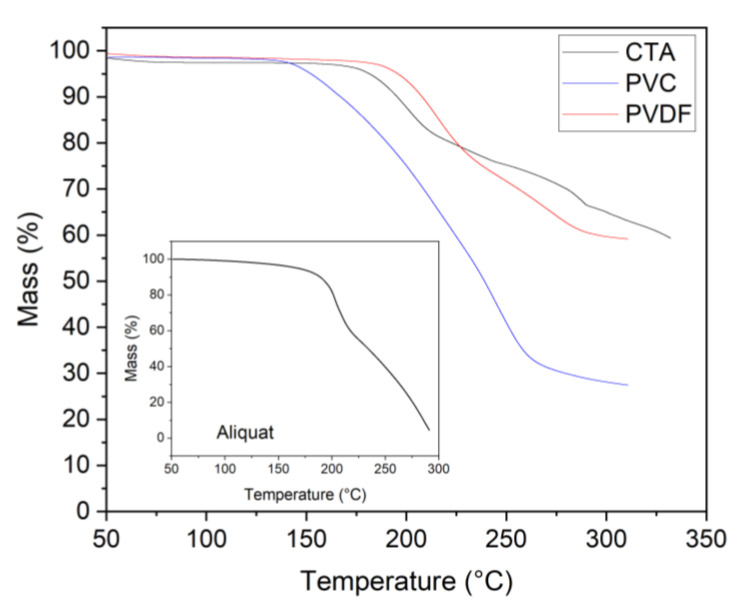
TG curves for PIMs containing 40 wt% Aliquat 336 and 60 wt% base polymer PVC, CTA, or PVDF-HFP (heating rate 10 K/min and nitrogen purge gas). Inset: Aliquat 336 TG curve.

**Figure 3:**
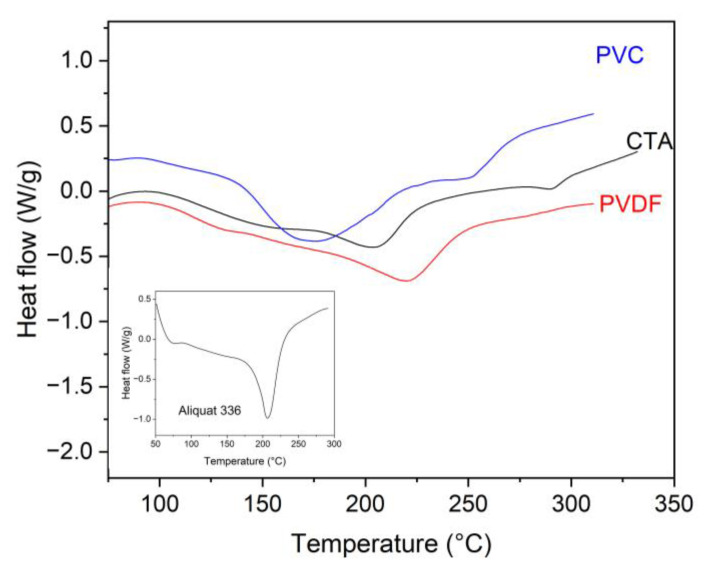
DTA curves for PIMs containing 40 wt% Aliquat 336 and 60 wt% base polymer PVC, CTA, or PVDF-HFP (heating rate: 10 K/min, nitrogen purge gas). Inset: Aliquat 336 DTA curve.

A combination of DTA and TGA was employed to investigate thermal behavior—particularly the separation/desorption of Aliquat 336 from PIMs containing PVC, CTA, or PVDF-HFP as their base polymer (Figure 2 and Figure 3).

The most striking feature of the TG curves is the markedly different temperatures at which the PIMs exhibit sharp mass loss, along with the varying steepness of the mass versus temperature curves (Figure 2).

Figure 2 clearly shows that the membrane with PVC as the base polymer releases its Aliquat 336 at the lowest temperature. The process of mass loss begins at about 140 °C and ends at 270 °C. Pure Aliquat 336 decomposes in the same temperature range (Figure 2, inset), indicating that the polymer matrix of the PVC retains Aliquat 336 poorly. A closer examination of the TG curve for this membrane shows that at a temperature of about 225 °C, decomposition of the polymer begins, which coincides with the literature data [27]. This is also the reason why the total mass loss of the sample exceeds the amount of extractant in the membrane, which is 40 wt.% for all membranes. At a slightly higher temperature (about 300 °C), the decomposition of the CTA polymer also takes place (Figure 2). In the CTA-based membrane, the extractant separates/decomposes in the range from 175 °C to 290 °C. Only PVDF-HFP is thermally stable up to 310 °C, and the liberated Aliquat 336 is exactly 40 wt.%, with its release temperature being the highest (Figure 2). This suggests that Aliquat 336 is most strongly “trapped” within the PVDF-HFP polymer matrix compared to the other two PIMs studied. The thermal results could suggest that the three polymers have different microstructures and, probably, different intermolecular free volumes, which could be expected to lead to different strengths of interactions between the extractant and the polymer matrix. Another important result from the TGA of the three PIMs is the presence of a two-stage separation process of Aliquat 336 in two of the base polymers (i.e., CTA and PVDF-HFP) used. This fact could be explained by the presence of different locations in the structure of the polymer matrix that are suitable/accessible for the accommodation of the extractant.

The DTA measurements provided additional information about the thermal behavior of the PIMs studied regarding the release of Aliquat 336 and the decomposition of the polymer (Figure 3).

Figure 3 presents the DTA curves of the PIMs with the three different base polymers used. It is noted that in the PVC-based membrane the first endothermic reaction associated with the release of Aliquat 336 starts at the lowest temperature compared to the other two membranes, in the range 140–220 °C with a maximum of about 170 °C. This may mean that the extractant included in this polymer is the most thermally unstable among the three PIMs studied. Notably, the temperature range of the endothermic peak associated with extractant release aligns with that observed in the TGA measurements. A second endothermic peak is also seen at about 250 °C, which is associated with the decomposition of the polymer. The DTA curve of the CTA-based membrane is different from that of the PVC-based one, with a maximum of the larger endothermic peak associated with the release of Aliquat 336 at about 200 °C. However, for this polymer, a broad endothermic peak is also observed at lower temperatures (120–170 °C). Notably, no visible lightening of the sample occurs within this range, indicating that the endothermic effect is not related to the release of Aliquat 336 but is more likely associated with some structural reorganization of the polymer (or phase transition), leading to increased mobility of the polymer chains. For this membrane, a distinct decomposition peak at approximately 285 °C is also observed, corresponding to the thermal degradation of CTA. The observed endothermic peak at about 200 °C for the PVDF-HFP-based membrane is within the temperature range of the mass decrease step obtained in the TGA measurements and is due to the release of Aliquat 336. In this case, the high-temperature peak is absent, reflecting the greater thermal stability of the polymer and the lack of decomposition within the studied temperature range. Similarly to the CTA-based membrane, this polymer exhibits a low-intensity endothermic peak in the 100–150 °C range, which is not accompanied by any change in membrane mass. As with CTA, this effect is most likely attributed to structural transformations within the PIM, such as a glass transition or other forms of structural relaxation. It is also worth emphasizing that membranes based on CTA and PVDF-HFP exhibit endothermic effects within low-temperature ranges, where no mass loss is observed, while also displaying significantly higher temperatures for Aliquat 336 release. This further supports the notion of improved retention within these membranes, likely due to stronger interactions with the polymer matrix or hindered extractant diffusion.

While requiring specific assumptions and bearing certain limitations, DTA in a combination with TGA can provide insights into the enthalpies of different reactions and structural transformations within the membranes studied during annealing. Determining the enthalpy of extractant release from various membranes provides critical insight and serves to validate the quantum-chemical predictions for the strength of interaction between the Aliquat 336 molecule and the polymer chains, discussed later. Since Aliquat 336 desorption involves low enthalpy change, the endothermic peaks observed before polymer degradation reflect most likely the polymer–Aliquat 336 de-bonding and extractant decomposition when reaching the membrane surface. Thus, the as-determined enthalpies of Aliquat 336 release from the various polymer matrices differ notably and, when related to the extractant content, can serve as an indicator of the strength of its interaction with each polymer. By comparing the measured enthalpy of the Aliquat 336 release from the PIMs with the enthalpy of the decomposition of pure Aliquat 336 (191 ± 10 J/g; Figure 3), it is possible to estimate the portion of the enthalpy change attributable to the interaction between Aliquat 336 and the polymer matrix. Assuming that the experimentally measured total enthalpy change primarily reflects the enthalpy of binding of the Aliquat to the polymer and the enthalpy of its decomposition, we subtracted the decomposition enthalpy of the pure Aliquat from the total thermal effect measured. This allowed us to isolate the portion of the enthalpy change attributable to binding. For PVDF-HFP-based PIMs, this interaction enthalpy was determined to be 18 ± 3 kcal/mol of Aliquat 336, a value closely matching the quantum chemical calculations outlined later. This strong agreement supports the conclusion that the observed enthalpy difference arises primarily from interactions between Aliquat 336 and the polymer chains. A lower enthalpy value was observed for the PVC-based PIMs (15 ± 2 kcal/mol), suggesting weaker interactions. In contrast, the enthalpy determination for CTA-based PIMs carries a larger uncertainty due to the complexity of the endothermic transitions and the difficulty in clearly isolating the peak associated with Aliquat 336 separation from the membrane.

### 3.2. Quantum Chemical Calculations

**Figure 4:**
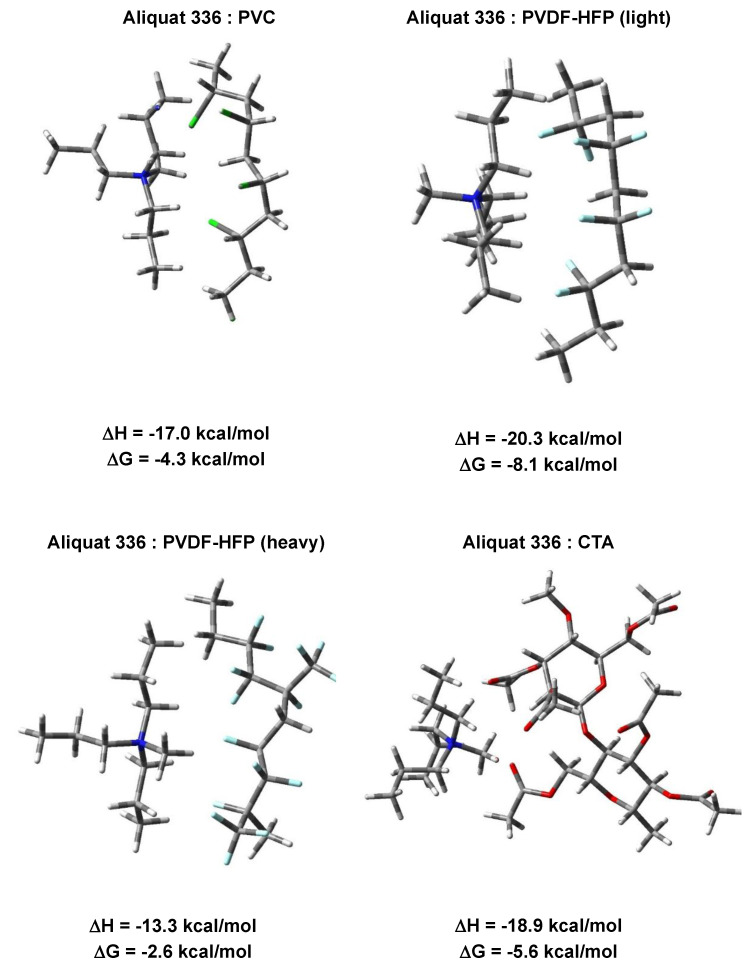
M062X fully optimized structures of *Aliquat 336 cation: Polymer* complexes and the respective formation ΔH and ΔG (in kcal/mol).

Fully optimized structures of *Aliquat 336 cation: Polymer* complexes are shown in Figure 4, along with the respective formation enthalpies and free energies. The calculations imply that the geometry of the interacting entities, which apparently is quite rigid, does not change significantly upon complex formation; as seen, they retain the overall shape characteristic of the individual reagents. It should be noted that several different positions of the attacking Aliquat 336 cation with respect to the host polymer were modeled and these structures, shown in Figure 1, represent the lowest energy (i.e., the most stable) constructs among them. The interactions between the positively charged Aliquat 336 cation and neutral polymeric fragments are of electrostatic origin (charge–dipole interactions) and, as the calculations imply, are all favorably characterized with negative ΔHs and ΔGs (Figure 4). This finding is in line with the experimental observations, which demonstrate that, indeed, the polymer membrane is able to bind the incoming Aliquat 336. Furthermore, the calculations predict noticeable differences in binding affinities of different polymeric materials: the “light”-chain PVDF-HFP exhibits the strongest preference toward Aliquat 336 (ΔH/ΔG = −20.3/−8.1 kcal/mol), followed by CTA (ΔH/ΔG = −18.9/−5.6 kcal/mol) and PVC (ΔH/ΔG = −17.0/−4.3 kcal/mol). These results reveal that the force of Aliquat 336 retention increases in the order PVC < CTA < PVDF-HFP, thus agreeing with the order of the Aliquat 336 retention strength by the three base polymers studied, already observed in the thermal analysis experiments. Unexpectedly, the co-polymer of PVDF-HFP, comprising alternating “light” and “heavy” fragments, is characterized with the highest (least favorable) energies of complex formation (ΔH/ΔG = −13.3/−2.6 kcal/mol). This is probably due to the steric hindrance imposed by the bulky substituents in the “heavy” chain of the co-polymer. Allegedly, the PVDF-HFP co-polymer behaves differently depending on the length, partial mass, and locality of the monomeric units interacting with the Aliquat 336 cation.

It should be noted that the arrangement of the enthalpies of interaction between the Aliquat 336 and the polymer monomers/chains, as well as the absolute values of the enthalpies themselves, obtained by thermal analysis, are in very good agreement with those obtained from theoretical calculations outlined above.

### 3.3. Aliquat 336 Leaching and TGA of the PIMs Post-Leaching

The thermal analysis results and the quantum chemical calculations clearly demonstrated that PVDF-HFP exhibited stronger interactions with Aliquat 336 compared to the other two base polymers. These findings were supported by the Aliquat 336 leaching experiments at two different NaCl concentrations of the aqueous solution, which showed that the amount of Aliquat 336 leached over a 24 h period decreased in the order PVC > CTA > PVDF-HFP (Table 1).

**Figure 5:**
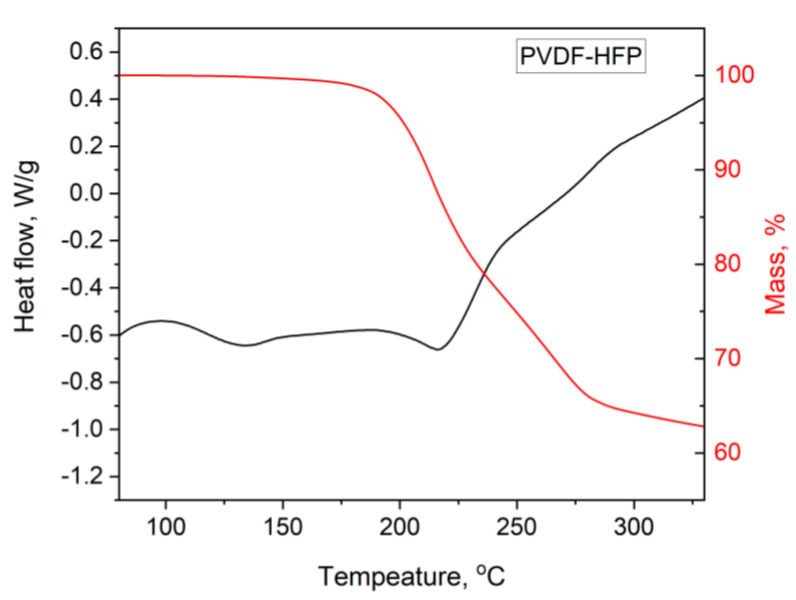
TGA and DTA curves of a PIM containing 40 wt% Aliquat 336 and 60 wt% PVDF-HFP after immersion in 0.1 M NaCl water solution for 24 h.

The TGA of the membranes after the leaching process (Figure 5) showed that the release temperature of Aliquat 336 in PVDF-HFP-based PIMs remained unchanged at approximately 200 °C, thus indicating that the Aliquat 336 lost during leaching experiment was bound to the polymer matrix as equally strongly as the one that stayed in the membrane. Also of interest is the distinct thermal peak in the 110–150 °C range (Figure 5), during which no mass loss is observed. The enthalpy associated with this peak, determined to be 60 J/g, can be attributed to solid-phase process within the membrane, most likely related to the de-bonding of a less strongly bound Aliquat 336 that is not immediately desorbed or to some structural reorganization of the polymer leading to increased mobility of the polymer chains.

The relatively good agreement between the percentage of Aliquat 336 lost due to leaching as determined by TGA (i.e., 9.6%) and by weighing the membrane before and after leaching (9.4%) in 0.1 M NaCl solution confirmed the possibility of using TGA for assessing PIM composition reported earlier [28]. For the other two membranes, such an assessment by TGA was difficult to perform due to the simultaneous decomposition of the polymers at higher temperatures.

## 4. Conclusions

A thermal analysis (TGA and DTA) approach for comparing PIM stability in terms of extractant retention of PIMs containing the same extractant but different base polymers was developed. This approach, applied to PIMs containing Aliquat 336 and one of the three most frequently used base polymers (PVC, CTA, or PVDF-HFP), showed that the PIM stability decreased in the order PVDF-HFP > CTA > PVC. This order was confirmed theoretically by quantum chemical (DFT) calculations considering the interactions between the extractant and the polymers’ monomers, which also identified the most probable interaction sites between them. An experimental validation of the thermal analysis approach was also provided by 24 h PIM leaching experiments in 0.1 M and 0.05 M NaCl aqueous solutions.

On the basis of the results obtained, it can be concluded that the thermal analysis approach, including TGA and DTA, developed in the present study allows for reliable comparison of the stability of PIMs containing the same extractant but different base polymers and is an attractive alternative to the time-consuming and labor-intensive conventional approach using multiple consecutive cycles of extraction and back-extraction steps involving the PIMs being compared.

## Figures and Tables

**Table 1:** Percentage mass loss ± standard deviation (MS ± SD) of the PIMs containing 40 wt.% Aliquat 336 and 60 wt.% PVC, CTA, or PVDF-HFP after immersion in 0.05 M and 0.1 M NaCl solutions for 24 h.

PIM Base Polymer	PVC	CTA	PVDF-HFP
MS ± SD [%] at 0.05 M NaCl	6.37 ± 0.35	5.85 ± 0.30	4.76 ± 0.30
MS ± SD [%] at 0.1 M NaCl	5.03 ± 0.30	4.02 ± 0.25	3.75 ± 0.20

## Data Availability

Data is contained within the article or Supplementary Materials.

## Supplementary Materials

The following supporting information can be downloaded at: https://www.mdpi.com/article/10.3390/membranes15100309/s1.
