# Supplementary material for: Assessing the Stability of Polymer Inclusion Membranes: The Case of Aliquat 336-Based Membranes
Source: Membranes (Basel). 2025 Oct 13;15(10):309. doi: 10.3390/membranes15100309 (PMC12565752; doi:10.3390/membranes15100309)
Supplement: Supplementary file 1 [file membranes-15-00309-s001.zip › membranes-3882976-supplementary.pdf]

Supporting information

Assessing the Stability of Polymer Inclusion Membranes: The Case of Aliquat 336-Based Membranes

Kalina Velikova [1], Todor Dudev [1], Tsveta Sarafska [1], Lea Kukoc Modun [2], Spas D. Kolev [1,3,4, *], Tony Spassov [1,5,*]

Optimized coordinates of the participating entities and the respective convergence criteria

Aliquat

1	6	0	-0.110232	1.071713	-0.425767
2	1	0	0.852159	1.554790	-0.606256
3	1	0	-0.553824	0.846290	-1.400656
4	6	0	0.262688	-0.204381	1.697031
5	1	0	-0.728256	0.007382	2.093018
6	1	0	0.617871	-1.165554	2.072424
7	1	0	0.951549	0.586713	1.989212
8	6	0	-0.834980	-1.319046	-0.210695
9	1	0	-0.646379	-1.511099	-1.271716
10	1	0	-0.581050	-2.226240	0.346637
11	6	0	1.532470	-0.774735	-0.337955
12	1	0	1.569777	-1.847840	-0.127263
13	1	0	1.471516	-0.645116	-1.423362
14	6	0	-0.995422	2.020540	0.375558
15	1	0	-0.467457	2.353232	1.274456
16	1	0	-1.917580	1.533703	0.701476
17	6	0	-2.301713	-0.969058	-0.003675
18	1	0	-2.580085	-0.107516	-0.618264
19	1	0	-2.503613	-0.712740	1.041292
20	6	0	2.776599	-0.094642	0.219445
21	1	0	2.692912	0.996311	0.167087
22	1	0	2.913670	-0.359933	1.271675

23	7	0	0.197193	-0.281560	0.205506
24	6	0	4.000791	-0.549399	-0.580322
25	1	0	4.908369	-0.107439	-0.166467
26	1	0	4.114433	-1.636717	-0.546740
27	1	0	3.925915	-0.245726	-1.628103
28	6	0	-1.336318	3.234650	-0.492159
29	1	0	-1.926535	3.954069	0.077425
30	1	0	-0.431981	3.744118	-0.837526
31	1	0	-1.919146	2.942398	-1.370546
32	6	0	-3.159447	-2.173758	-0.402500
33	1	0	-2.936600	-3.044119	0.220866
34	1	0	-4.218290	-1.939362	-0.283181
35	1	0	-2.994335	-2.449990	-1.447799

Item	Value	Threshold	Converged?
Maximum Force	0.000036	0.000450	YES
RMS Force	0.000008	0.000300	YES
Maximum Displacement	0.001274	0.001800	YES
RMS Displacement	0.000255	0.001200	YES

PVC-tetramer

1	6	0	4.863950	1.181256	-1.015055
2	1	0	4.518195	2.213811	-1.099398
3	1	0	5.951829	1.192399	-0.911474
4	1	0	4.618632	0.664219	-1.948471
5	6	0	4.221271	0.479134	0.176436
6	1	0	4.634795	-0.532893	0.273012
7	1	0	4.451619	1.010892	1.108156
8	6	0	2.709940	0.312007	0.066121
9	6	0	2.124138	-0.429647	1.268706
10	6	0	0.846616	-1.227100	1.007140
11	6	0	-0.323221	-0.400762	0.486358
12	1	0	-0.421035	0.451060	1.170696
13	1	0	-0.102130	0.028188	-0.494619
14	6	0	-1.662635	-1.146579	0.459311

15	6	0	-2.892537	-0.303179	0.811351
16	6	0	-3.326768	0.778397	-0.171997
17	6	0	-4.710260	1.313673	0.160962
18	1	0	-4.993202	2.110943	-0.528650
19	1	0	-5.444204	0.505580	0.080922
20	1	0	-4.734928	1.711452	1.179844
21	1	0	2.463530	-0.211009	-0.863173
22	1	0	1.931442	0.286284	2.076593
23	1	0	2.879406	-1.133402	1.635228
24	1	0	0.554536	-1.713101	1.943058
25	1	0	-1.624933	-1.978529	1.169397
26	1	0	-3.740721	-0.991157	0.909342
27	1	0	-2.728395	0.150263	1.797603
28	1	0	-3.302351	0.388148	-1.190919
29	17	0	-2.158171	2.167565	-0.177832
30	17	0	-1.948113	-1.945505	-1.142979
31	17	0	1.239898	-2.607559	-0.103980
32	17	0	1.924324	1.944537	-0.093041

Item	Value	Threshold	Converged?
Maximum Force	0.000011	0.000450	YES
RMS Force	0.000002	0.000300	YES
Maximum Displacement	0.001024	0.001800	YES
RMS Displacement	0.000171	0.001200	YES

PVDF-dimer

1	6	0	6.458325	-0.855359	0.431048
2	1	0	6.533316	-0.317636	1.378834
3	1	0	7.169075	-1.683759	0.451625
4	1	0	6.744908	-0.177248	-0.375124
5	6	0	5.044584	-1.395524	0.220184
6	1	0	4.961910	-1.921467	-0.735740
7	1	0	4.768458	-2.094155	1.013377
8	6	0	4.027513	-0.273765	0.220825
9	6	0	2.592930	-0.752298	-0.103341

10	6	0	1.366115	0.180777	0.151738
11	9	0	4.006542	0.347558	1.427892
12	9	0	4.358488	0.654766	-0.716736
13	9	0	2.588205	-1.131723	-1.405577
14	9	0	2.364771	-1.865635	0.641872
15	9	0	1.262880	0.322849	1.509936
16	6	0	1.539205	1.602186	-0.418567
17	9	0	1.752167	1.541659	-1.738675
18	9	0	0.441652	2.325286	-0.199197
19	9	0	2.553510	2.241373	0.157337
20	6	0	0.140340	-0.563995	-0.397496
21	1	0	0.269168	-0.738231	-1.467408
22	1	0	0.133983	-1.536641	0.101535
23	6	0	-1.233976	0.068178	-0.202432
24	6	0	-2.336928	-1.016706	-0.252348
25	6	0	-3.825312	-0.595196	-0.361986
26	6	0	-4.713439	-1.820421	-0.221508
27	1	0	-5.731260	-1.539048	-0.491938
28	1	0	-4.362086	-2.601809	-0.896212
29	1	0	-4.698090	-2.191520	0.804494
30	9	0	-1.502706	0.963259	-1.182595
31	9	0	-1.341575	0.692276	0.991533
32	9	0	-2.081026	-1.794822	-1.336622
33	9	0	-2.185145	-1.794332	0.847108
34	9	0	-3.994063	-0.050328	-1.613294
35	6	0	-4.246771	0.497405	0.637709
36	9	0	-3.692068	1.673475	0.359722
37	9	0	-3.917915	0.143019	1.885537
38	9	0	-5.576483	0.659076	0.593271

Item	Value	Threshold	Converged?
Maximum Force	0.000012	0.000450	YES
RMS Force	0.000003	0.000300	YES
Maximum Displacement	0.001037	0.001800	YES
RMS Displacement	0.000218	0.001200	YES

PVDF-tetramer-light

1	6	0	4.397090	-2.091532	0.309347
2	1	0	3.884926	-2.854976	-0.279941
3	1	0	5.472698	-2.266702	0.238571
4	1	0	4.099117	-2.206502	1.353847
5	6	0	4.058933	-0.692485	-0.199066
6	1	0	4.588464	0.070936	0.379954
7	1	0	4.353780	-0.574452	-1.247507
8	6	0	2.579136	-0.384233	-0.101545
9	6	0	2.176218	0.965216	-0.671824
10	6	0	0.807223	1.494082	-0.255127
11	6	0	-0.354277	0.523144	-0.399910
12	1	0	-0.469585	0.292118	-1.462057
13	1	0	-0.113947	-0.408978	0.115294
14	6	0	-1.679555	1.021160	0.159552
15	6	0	-2.912588	0.224692	-0.255390
16	6	0	-2.808455	-1.285313	-0.118624
17	6	0	-4.120581	-2.022177	-0.212995
18	1	0	-3.925083	-3.094606	-0.160315
19	1	0	-4.768869	-1.730249	0.615016
20	1	0	-4.611980	-1.789591	-1.159950
21	1	0	2.202594	0.890055	-1.762149
22	1	0	2.901693	1.724072	-0.364848
23	1	0	-3.737846	0.589773	0.362470
24	1	0	-3.138796	0.453869	-1.300283
25	9	0	2.181167	-0.473732	1.206458
26	9	0	1.873016	-1.366539	-0.764472
27	9	0	0.883114	1.953736	1.025067
28	9	0	0.583291	2.599129	-1.039422
29	9	0	-1.618942	1.047252	1.522234
30	9	0	-1.912724	2.313904	-0.228886
31	9	0	-1.967668	-1.759784	-1.103161
32	9	0	-2.198591	-1.603977	1.065002

Item	Value	Threshold	Converged?
Maximum Force	0.000005	0.000450	YES
RMS Force	0.000001	0.000300	YES
Maximum Displacement	0.000205	0.001800	YES
RMS Displacement	0.000047	0.001200	YES

CTA-dimer

1	6	0	0.742302	0.623963	0.111536
2	6	0	1.772080	1.056274	1.151793
3	6	0	3.447824	-0.272108	-0.193857
4	6	0	2.302821	-0.590924	-1.158845
5	1	0	1.524505	2.054278	1.523627
6	1	0	0.718213	1.368366	-0.705606
7	1	0	3.500029	-1.075700	0.554794
8	1	0	2.255990	0.199950	-1.925355
9	8	0	-0.483229	0.522853	0.744586
10	6	0	-1.613909	0.413254	-0.102789
11	6	0	-2.429634	-0.789257	0.353910
12	6	0	-2.478749	1.676165	-0.009015
13	1	0	-1.299350	0.254159	-1.144436
14	6	0	-3.736627	-0.871836	-0.413788
15	1	0	-2.623969	-0.709623	1.429578
16	1	0	-2.754990	1.841755	1.045604
17	6	0	-4.487938	0.463648	-0.331678
18	1	0	-3.551976	-1.128722	-1.462257
19	1	0	-4.740027	0.651990	0.725840
20	6	0	-1.737820	2.887939	-0.521016
21	1	0	-0.773538	2.987173	-0.013255
22	1	0	-1.570797	2.809838	-1.601185
23	8	0	-2.532895	4.039668	-0.243662
24	6	0	-1.992207	5.213622	-0.621412
25	8	0	-0.911318	5.296277	-1.154537
26	6	0	-2.900867	6.364878	-0.291376
27	1	0	-2.439904	7.293134	-0.623231

28	1	0	-3.866074	6.222496	-0.782466
29	1	0	-3.076950	6.394490	0.786400
30	8	0	-3.636425	1.498522	-0.800781
31	6	0	-5.743366	0.480869	-1.179613
32	1	0	-6.449197	-0.279831	-0.837028
33	1	0	-6.220099	1.460643	-1.107704
34	1	0	-5.495453	0.278468	-2.224745
35	8	0	-4.517661	-1.898511	0.200204
36	6	0	-5.153368	-2.781818	-0.605286
37	8	0	-5.137667	-2.720609	-1.808741
38	6	0	-5.872044	-3.819086	0.211856
39	1	0	-6.369491	-4.520576	-0.454954
40	1	0	-5.157416	-4.341550	0.851763
41	1	0	-6.602564	-3.331256	0.861727
42	8	0	-1.675900	-1.969852	0.097745
43	6	0	-1.001082	-2.518464	1.132380
44	8	0	-1.136139	-2.171056	2.281181
45	6	0	-0.058514	-3.585261	0.661019
46	1	0	0.889004	-3.085292	0.433133
47	1	0	0.093453	-4.310358	1.460471
48	1	0	-0.423938	-4.067439	-0.246845
49	8	0	1.077278	-0.635836	-0.437939
50	6	0	2.492237	-1.906355	-1.878835
51	1	0	1.571646	-2.187378	-2.398264
52	1	0	3.307008	-1.826278	-2.602003
53	8	0	2.821125	-2.909868	-0.910534
54	6	0	2.960219	-4.161649	-1.394113
55	8	0	2.808011	-4.435656	-2.558648
56	6	0	3.327547	-5.129575	-0.302774
57	1	0	2.600074	-5.069885	0.510127
58	1	0	3.358078	-6.137411	-0.712297
59	1	0	4.305425	-4.862753	0.106143
60	8	0	4.625600	-0.207088	-0.958228
61	6	0	5.809149	-0.445784	-0.215446

62	1	0	6.637568	-0.397358	-0.922627
63	1	0	5.958479	0.319045	0.554307
64	1	0	5.776972	-1.443235	0.242607
65	8	0	1.769367	0.117434	2.217572
66	6	0	1.061120	0.453423	3.334994
67	8	0	0.556878	1.534832	3.492208
68	6	0	1.015987	-0.695347	4.296825
69	1	0	0.669872	-0.337200	5.264834
70	1	0	0.316515	-1.437414	3.899877
71	1	0	2.000316	-1.159110	4.382699
72	6	0	3.168659	1.050299	0.533223
73	1	0	3.920094	1.249708	1.301282
74	8	0	3.173197	2.106550	-0.431055
75	6	0	4.331041	2.792322	-0.599669
76	8	0	5.302576	2.633986	0.095581
77	6	0	4.215158	3.754503	-1.746642
78	1	0	3.291271	4.330664	-1.666061
79	1	0	5.082069	4.412342	-1.753440
80	1	0	4.176665	3.184531	-2.678960

Item	Value	Threshold	Converged?
Maximum Force	0.000006	0.000450	YES
RMS Force	0.000001	0.000300	YES
Maximum Displacement	0.001539	0.001800	YES
RMS Displacement	0.000234	0.001200	YES

PVC-tetramer-Aliquat

1	6	0	5.286185	-1.182356	-1.044960
2	1	0	4.790452	-1.245657	-2.016678
3	1	0	6.361499	-1.280376	-1.207816
4	1	0	4.962265	-2.032654	-0.434917
5	6	0	4.969112	0.134669	-0.343935
6	1	0	5.515993	0.183734	0.605735
7	1	0	5.302142	0.985018	-0.949960
8	6	0	3.491713	0.311841	-0.009993

9	6	0	3.241880	1.562570	0.825102
10	6	0	1.839014	1.804473	1.397057
11	6	0	0.789762	2.264375	0.387741
12	1	0	1.287751	3.013779	-0.242191
13	1	0	0.511621	1.462953	-0.290626
14	6	0	-0.453686	2.939067	0.968238
15	6	0	-1.166466	3.908042	0.014253
16	6	0	-2.085229	3.343917	-1.063386
17	6	0	-2.692985	4.446886	-1.913668
18	1	0	-3.358837	4.029895	-2.670874
19	1	0	-3.272529	5.121523	-1.275968
20	1	0	-1.913005	5.024378	-2.416844
21	1	0	3.121248	-0.572775	0.517830
22	1	0	3.528314	2.448971	0.245533
23	1	0	3.931633	1.499601	1.674600
24	1	0	1.931724	2.555383	2.186723
25	1	0	-0.176173	3.499456	1.865552
26	1	0	-1.798261	4.568347	0.620201
27	1	0	-0.402292	4.540288	-0.454594
28	1	0	-2.867226	2.738363	-0.603466
29	17	0	-1.206369	2.184821	-2.160990
30	17	0	-1.661876	1.718448	1.577463
31	17	0	1.313405	0.306967	2.299885
32	17	0	2.532939	0.359288	-1.560973
33	6	0	-1.868071	-3.336556	-0.611891
34	1	0	-1.150610	-3.984961	-1.119502
35	1	0	-2.387682	-3.942534	0.137505
36	6	0	-0.752180	-1.093596	-0.630743
37	1	0	-1.664680	-0.523002	-0.793029
38	1	0	-0.042173	-0.498640	-0.055636
39	1	0	-0.312654	-1.378728	-1.586146
40	6	0	-1.766770	-1.920846	1.455473
41	1	0	-1.715343	-2.801125	2.104908
42	1	0	-1.151898	-1.128001	1.894189

43	6	0	0.249916	-2.968876	0.608233
44	1	0	0.649246	-2.332977	1.403804
45	1	0	-0.031966	-3.932786	1.045339
46	6	0	-2.844901	-2.781739	-1.643697
47	1	0	-2.292417	-2.314075	-2.464089
48	1	0	-3.490867	-2.013697	-1.212379
49	6	0	-3.209537	-1.454036	1.334205
50	1	0	-3.844691	-2.254835	0.942025
51	1	0	-3.285759	-0.598669	0.654685
52	6	0	1.304111	-3.148679	-0.475876
53	1	0	0.897682	-3.674322	-1.347005
54	1	0	1.659594	-2.173413	-0.823677
55	7	0	-1.055009	-2.319853	0.171868
56	6	0	-3.711724	-1.045897	2.722254
57	1	0	-4.743434	-0.694740	2.665165
58	1	0	-3.683328	-1.890535	3.416982
59	1	0	-3.103565	-0.239747	3.142944
60	6	0	-3.700251	-3.926687	-2.190055
61	1	0	-4.371078	-3.562489	-2.969834
62	1	0	-3.079288	-4.715175	-2.625729
63	1	0	-4.313011	-4.374126	-1.401717
64	6	0	2.480932	-3.943462	0.095419
65	1	0	3.261086	-4.060157	-0.659059
66	1	0	2.920465	-3.430779	0.957469
67	1	0	2.172476	-4.942304	0.417303

Item	Value	Threshold	Converged?
Maximum Force	0.000023	0.000450	YES
RMS Force	0.000005	0.000300	YES
Maximum Displacement	0.001161	0.001800	YES
RMS Displacement	0.000251	0.001200	YES

PVDF-dimer-Aliquat

1	6	0	-4.619379	3.740622	1.496809
2	1	0	-5.314378	3.282947	2.203696

3	1	0	-4.289649	4.694027	1.913439
4	1	0	-5.152985	3.938784	0.565475
5	6	0	-3.410202	2.835805	1.259063
6	1	0	-2.718626	3.285418	0.538881
7	1	0	-2.865406	2.657274	2.189396
8	6	0	-3.837859	1.491094	0.710911
9	6	0	-2.653547	0.557425	0.367023
10	6	0	-2.920089	-0.938668	0.039543
11	9	0	-4.621888	0.840060	1.605363
12	9	0	-4.556133	1.657491	-0.431017
13	9	0	-1.989935	1.110522	-0.686565
14	9	0	-1.814487	0.571758	1.431182
15	9	0	-3.268646	-1.550243	1.212120
16	6	0	-4.104473	-1.162712	-0.936031
17	9	0	-3.914929	-0.451556	-2.053587
18	9	0	-4.162855	-2.456387	-1.276646
19	9	0	-5.264415	-0.837490	-0.390612
20	6	0	-1.702892	-1.654507	-0.568730
21	1	0	-1.983722	-2.707287	-0.638668
22	1	0	-1.526922	-1.286365	-1.583326
23	6	0	-0.389849	-1.586171	0.189320
24	6	0	0.651833	-2.584407	-0.389458
25	6	0	1.916270	-2.797384	0.483635
26	6	0	1.718659	-3.846342	1.559873
27	1	0	2.587998	-3.833554	2.217898
28	1	0	0.820411	-3.632897	2.137460
29	1	0	1.627203	-4.835100	1.106303
30	9	0	-0.546827	-1.845397	1.504213
31	9	0	0.177048	-0.345007	0.077533
32	9	0	0.053594	-3.780724	-0.573230
33	9	0	0.982524	-2.104382	-1.617370
34	9	0	2.229896	-1.575398	1.062800
35	6	0	3.133573	-3.141459	-0.400010
36	9	0	3.528102	-2.083989	-1.124919

37	9	0	2.840450	-4.142667	-1.227481
38	9	0	4.168471	-3.502548	0.361235
39	6	0	2.834028	1.536447	0.987576
40	1	0	1.996092	0.841761	1.096313
41	1	0	2.656590	2.389226	1.647681
42	6	0	3.849963	3.044029	-0.742940
43	1	0	4.777754	2.468297	-0.707711
44	1	0	3.701528	3.353294	-1.780669
45	6	0	3.936159	4.252809	0.176408
46	1	0	2.977433	4.781859	0.211637
47	1	0	4.172716	3.937665	1.198194
48	7	0	2.734334	2.072650	-0.430829
49	6	0	1.366880	2.727091	-0.555375
50	1	0	0.642959	1.910132	-0.478053
51	1	0	1.247636	3.361038	0.327091
52	6	0	4.142589	0.850151	1.351094
53	1	0	4.991202	1.528201	1.211936
54	1	0	4.306281	-0.028663	0.720092
55	6	0	2.827821	0.933945	-1.399711
56	1	0	2.078093	0.187838	-1.137420
57	1	0	2.655858	1.310977	-2.407006
58	1	0	3.823250	0.497382	-1.340852
59	6	0	1.126375	3.544557	-1.817806
60	1	0	1.798379	4.408252	-1.849831
61	1	0	1.313836	2.948710	-2.716803
62	6	0	4.085628	0.415578	2.818249
63	1	0	3.250439	-0.267505	2.993059
64	1	0	5.006923	-0.098645	3.097278
65	1	0	3.969168	1.277680	3.481689
66	6	0	5.024566	5.202899	-0.328491
67	1	0	5.115614	6.065995	0.332789
68	1	0	5.998024	4.705460	-0.365293
69	1	0	4.793049	5.571461	-1.332085
70	6	0	-0.323490	4.036458	-1.831271

71	1	0	-0.509892	4.644821	-2.717793
72	1	0	-1.025986	3.199076	-1.841248
73	1	0	-0.536321	4.653944	-0.953007

Item	Value	Threshold	Converged?
Maximum Force	0.000023	0.000450	YES
RMS Force	0.000004	0.000300	YES
Maximum Displacement	0.001733	0.001800	YES
RMS Displacement	0.000401	0.001200	YES

PVDF-tetramer-light-Aliquat

1	6	0	3.405671	-3.476473	1.566641
2	1	0	2.768463	-3.189415	2.406665
3	1	0	3.938627	-4.387767	1.843114
4	1	0	4.144029	-2.688756	1.402387
5	6	0	2.574788	-3.721484	0.309183
6	1	0	3.213882	-4.008581	-0.532432
7	1	0	1.858644	-4.533872	0.468729
8	6	0	1.787718	-2.499887	-0.111222
9	6	0	0.989201	-2.668527	-1.394668
10	6	0	-0.027401	-1.575822	-1.713871
11	6	0	-1.305792	-1.605416	-0.886804
12	1	0	-1.762818	-2.593619	-0.994569
13	1	0	-1.041741	-1.482654	0.164449
14	6	0	-2.352087	-0.563290	-1.252504
15	6	0	-3.634293	-0.589254	-0.429135
16	6	0	-3.467341	-0.546663	1.080856
17	6	0	-4.729838	-0.281462	1.856162
18	1	0	-4.503619	-0.319351	2.923092
19	1	0	-5.125125	0.703405	1.601745
20	1	0	-5.476341	-1.042404	1.620465
21	1	0	0.467532	-3.629425	-1.368665
22	1	0	1.698736	-2.698171	-2.226521
23	1	0	-4.226955	0.271081	-0.753341
24	1	0	-4.191353	-1.495396	-0.683229

25	6	0	0.426237	1.342976	1.405933
26	1	0	-0.415718	1.870372	1.859411
27	1	0	0.026932	0.649483	0.659169
28	6	0	0.344691	2.871166	-0.530134
29	1	0	0.928023	3.642671	-1.038242
30	1	0	0.232517	2.013543	-1.200719
31	6	0	2.460373	1.736353	0.065913
32	1	0	3.078597	1.487561	0.932139
33	1	0	2.134768	0.802273	-0.397059
34	6	0	-1.020643	3.428482	-0.148411
35	1	0	-0.920027	4.241291	0.579263
36	1	0	-1.643463	2.648507	0.296172
37	6	0	3.267329	2.569536	-0.921370
38	1	0	2.710545	2.710917	-1.851625
39	1	0	3.492145	3.561424	-0.513625
40	7	0	1.205918	2.385300	0.622253
41	9	0	2.647107	-1.432541	-0.252802
42	9	0	0.943423	-2.128481	0.916975
43	9	0	0.583107	-0.346199	-1.571849
44	9	0	-0.326969	-1.689292	-3.035722
45	9	0	-1.798379	0.694479	-1.157297
46	9	0	-2.723638	-0.700476	-2.555307
47	9	0	-2.924600	-1.738278	1.503946
48	9	0	-2.523113	0.407072	1.410020
49	6	0	4.576130	1.836902	-1.228630
50	1	0	5.156313	2.385778	-1.972331
51	1	0	5.191952	1.731852	-0.330796
52	1	0	4.379441	0.836374	-1.624865
53	6	0	-1.706694	3.959902	-1.409627
54	1	0	-1.831899	3.164195	-2.149012
55	1	0	-2.695567	4.353848	-1.167587
56	1	0	-1.128707	4.767844	-1.867864
57	6	0	1.571134	3.531914	1.509417
58	1	0	1.940142	4.354415	0.897414

59	1	0	0.689345	3.852018	2.062783
60	1	0	2.346921	3.213875	2.204653
61	6	0	1.208415	0.592617	2.475748
62	1	0	1.943171	-0.073196	2.015058
63	1	0	1.745743	1.288289	3.130308
64	6	0	0.231045	-0.235029	3.313858
65	1	0	-0.346274	-0.918520	2.685252
66	1	0	0.772048	-0.831213	4.051273
67	1	0	-0.474630	0.408433	3.847484

Item	Value	Threshold	Converged?
Maximum Force	0.000083	0.000450	YES
RMS Force	0.000010	0.000300	YES
Maximum Displacement	0.000092	0.001800	YES
RMS Displacement	0.000044	0.001200	YES

CTA-dimer-Aliquat

1	6	0	0.865418	0.644199	-0.547865
2	6	0	-0.286044	1.442535	0.052456
3	6	0	0.794704	3.463375	-0.994536
4	6	0	1.896135	2.524369	-1.502540
5	1	0	-1.190415	0.829706	0.081474
6	1	0	0.592576	0.339123	-1.575961
7	1	0	1.102713	3.850950	-0.011577
8	1	0	1.616491	2.160124	-2.504098
9	8	0	1.082907	-0.452751	0.263449
10	6	0	1.856691	-1.492005	-0.316100
11	6	0	2.960094	-1.881322	0.655972
12	6	0	0.974968	-2.712309	-0.607137
13	1	0	2.322228	-1.142906	-1.249225
14	6	0	3.721888	-3.081814	0.119124
15	1	0	2.527647	-2.106355	1.637736
16	1	0	0.500736	-3.042362	0.333546
17	6	0	2.755648	-4.222886	-0.233579
18	1	0	4.297640	-2.800227	-0.769317

19	1	0	2.253250	-4.548339	0.693035
20	6	0	-0.105765	-2.356618	-1.601655
21	1	0	-0.674332	-1.495721	-1.238787
22	1	0	0.331292	-2.123805	-2.579806
23	8	0	-0.989015	-3.470949	-1.749918
24	6	0	-2.206884	-3.208527	-2.236864
25	8	0	-2.600574	-2.082873	-2.474972
26	6	0	-3.003403	-4.465002	-2.439633
27	1	0	-4.032687	-4.211101	-2.687228
28	1	0	-2.560327	-5.036348	-3.259646
29	1	0	-2.958629	-5.089610	-1.545256
30	8	0	1.772823	-3.738288	-1.146141
31	6	0	3.452742	-5.395040	-0.891779
32	1	0	4.189717	-5.831527	-0.213725
33	1	0	2.718969	-6.161060	-1.150448
34	1	0	3.967524	-5.068967	-1.798991
35	8	0	4.607756	-3.511790	1.148601
36	6	0	5.887093	-3.811598	0.794966
37	8	0	6.282189	-3.774948	-0.341301
38	6	0	6.695157	-4.192090	2.001939
39	1	0	7.713639	-4.419863	1.694262
40	1	0	6.689253	-3.371169	2.722448
41	1	0	6.243524	-5.061277	2.486240
42	8	0	3.862781	-0.787595	0.774399
43	6	0	3.671271	0.076255	1.798025
44	8	0	2.871594	-0.108329	2.684812
45	6	0	4.552263	1.281625	1.662005
46	1	0	3.998780	2.007677	1.056026
47	1	0	4.741989	1.702601	2.649083
48	1	0	5.484857	1.037398	1.152500
49	8	0	2.035593	1.422943	-0.608884
50	6	0	3.236360	3.212181	-1.623433
51	1	0	4.015766	2.476508	-1.842241
52	1	0	3.208461	3.954445	-2.424608

53	8	0	3.518622	3.859267	-0.379731
54	6	0	4.722285	4.479969	-0.316214
55	8	0	5.503068	4.471425	-1.232678
56	6	0	4.920383	5.156518	1.011084
57	1	0	4.723797	4.455424	1.825220
58	1	0	5.938914	5.534994	1.070946
59	1	0	4.212941	5.984610	1.104506
60	8	0	0.678285	4.496122	-1.934334
61	6	0	-0.091197	5.601555	-1.504025
62	1	0	0.037078	6.384668	-2.250911
63	1	0	-1.158031	5.352263	-1.434721
64	1	0	0.260070	5.967287	-0.530282
65	8	0	0.070177	1.840432	1.367675
66	6	0	-0.434618	1.120557	2.400310
67	8	0	-1.303063	0.289098	2.254883
68	6	0	0.214787	1.500115	3.692267
69	1	0	-0.370950	1.111272	4.523284
70	1	0	1.215171	1.054043	3.698363
71	1	0	0.323765	2.583791	3.761038
72	6	0	-0.517769	2.697898	-0.790806
73	1	0	-1.288621	3.320613	-0.327879
74	8	0	-0.963980	2.270910	-2.089430
75	6	0	-2.238646	1.877047	-2.214194
76	8	0	-3.033388	1.922530	-1.293739
77	6	0	-2.539315	1.329494	-3.576321
78	1	0	-2.404541	0.241754	-3.532937
79	1	0	-3.580122	1.536016	-3.825702
80	1	0	-1.868230	1.743554	-4.327897
81	6	0	-5.623995	0.192021	-0.388667
82	1	0	-6.620371	0.419087	-0.008551
83	1	0	-5.146093	1.123418	-0.699138
84	6	0	-5.512202	-1.252578	1.728303
85	1	0	-5.315297	-2.257507	1.348766
86	1	0	-4.995967	-1.162359	2.689737

87	6	0	-4.313358	0.931987	1.580513
88	1	0	-3.615078	0.547365	2.327912
89	1	0	-3.737143	1.521203	0.861301
90	6	0	-5.684315	-0.734551	-1.600389
91	1	0	-6.218672	-0.149585	-2.358000
92	1	0	-4.678732	-0.890640	-2.004118
93	6	0	-5.381304	1.789085	2.241771
94	1	0	-6.159987	2.077893	1.527962
95	1	0	-5.862365	1.241112	3.058085
96	7	0	-4.794302	-0.284620	0.799075
97	6	0	-7.015811	-1.077927	1.910513
98	1	0	-7.277210	-0.040002	2.129581
99	1	0	-7.536892	-1.355218	0.990783
100	6	0	-3.550408	-0.963612	0.305995
101	1	0	-3.055899	-0.294948	-0.399362
102	1	0	-2.900306	-1.158606	1.160398
103	1	0	-3.824910	-1.885176	-0.203781
104	6	0	-7.487219	-1.980314	3.052988
105	1	0	-8.570624	-1.916971	3.167921
106	1	0	-7.232378	-3.027351	2.862720
107	1	0	-7.029940	-1.687873	4.002868
108	6	0	-4.714855	3.046910	2.805525
109	1	0	-5.443545	3.668540	3.328826
110	1	0	-3.923197	2.787036	3.515111
111	1	0	-4.271193	3.645888	2.004828
112	6	0	-6.399620	-2.074860	-1.433025
113	1	0	-5.912523	-2.729022	-0.702045
114	1	0	-7.441819	-1.943278	-1.128832
115	1	0	-6.404819	-2.608337	-2.386433

Item	Value	Threshold	Converged?
Maximum Force	0.000008	0.000450	YES
RMS Force	0.000001	0.000300	YES
Maximum Displacement	0.000785	0.001800	YES
RMS Displacement	0.000172	0.001200	YES
